# Evaluation of the Feasibility of In Vitro Metabolic Interruption of Trimethylamine with Resveratrol Butyrate Esters and Its Purified Monomers

**DOI:** 10.3390/molecules29020429

**Published:** 2024-01-16

**Authors:** Ping-Hsiu Huang, De-Quan Chen, Yu-Wei Chen, Ming-Kuei Shih, Bao-Hong Lee, You-Lin Tain, Chang-Wei Hsieh, Chih-Yao Hou

**Affiliations:** 1 School of Food, Jiangsu Food and Pharmaceutical Science College, No. 4, Meicheng Road, Higher Education Park, Huai’an 223003, China; hugh0530@gmail.com; 2 Department of Seafood Science, College of Hydrosphere, National Kaohsiung, University of Science and Technology, Kaohsiung 81157, Taiwan; q3w2e24@gmail.com; 3 Department of Food Science and Biotechnology, National Chung Hsing University, Taichung 40227, Taiwan; naosa720928@gmail.com (Y.-W.C.); welson@nchu.edu.tw (C.-W.H.); 4 Department of Pediatrics, Kaohsiung Chang Gung Memorial Hospital, Kaohsiung 83301, Taiwan; tainyl@hotmail.com; 5 Graduate Institute of Food Culture and Innovation, National Kaohsiung University of Hospitality and Tourism, Kaohsiung 812301, Taiwan; mkshih@mail.nkuht.edu.tw; 6 Department of Horticulture, National Chiayi University, Chiayi 60004, Taiwan; bhlee@mail.ncyu.edu.tw; 7 Institute for Translational Research in Biomedicine, Kaohsiung Chang Gung Memorial Hospital, Kaohsiung 83301, Taiwan; 8 College of Medicine, Chang Gung University, Taoyuan 33305, Taiwan; 9 Department of Medical Research, China Medical University Hospital, Taichung 40447, Taiwan

**Keywords:** probiotic, trimethylamine-*N*-oxide (TMAO), short-chain fatty acid (SCFA), metabolism, co-cultured

## Abstract

Resveratrol (RSV), obtained from dietary sources, has been shown to reduce trimethylamine oxide (TMAO) levels in humans, and much research indicates that TMAO is recognized as a risk factor for cardiovascular disease. Therefore, this study investigated the effects of RSV and RSV-butyrate esters (RBE) on the proliferation of co-cultured bacteria and HepG2 cell lines, respectively, and also investigated the changes in trimethylamine (TMA) and TMOA content in the medium and flavin-containing monooxygenase-3 (FMO3) gene expression. This study revealed that 50 µg/mL of RBE could increase the population percentage of *Bifidobacterium longum* at a rate of 53%, while the rate was 48% for *Clostridium asparagiforme*. In contrast, co-cultivation of the two bacterial strains effectively reduced TMA levels from 561 ppm to 449 ppm. In addition, regarding TMA-induced HepG2 cell lines, treatment with 50 μM each of RBE, 3,4′-di-O-butanoylresveratrol (ED2), and 3-O-butanoylresveratrol (ED4) significantly reduced FMO3 gene expression from 2.13 to 0.40–1.40, which would also contribute to the reduction of TMAO content. This study demonstrated the potential of RBE, ED2, and ED4 for regulating TMA metabolism in microbial co-cultures and cell line cultures, which also suggests that the resveratrol derivative might be a daily dietary supplement that will be beneficial for health promotion in the future.

## 1. Introduction

CVD biomarkers like trimethylamine N-oxide (TMAO) are effective early detectors [1]. Recent research has linked TMAO to common CVD [2]. The gut microbiota converts phosphatidylcholine and L-carnitine into TMAO, a dietary metabolite. The gut microbiota converts choline or L-carnitine into TMA, which FMO3 in the liver converts to TMAO [3]. The microbial enzyme trimethylamine dehydrogenase (TMADH) breaks down TMA into DMA and formaldehyde [4]. The kidneys eliminate most TMAO, and TMAO reductase converts the rest to TMA [5]. Changes in gut microbiota raise TMAO levels, increasing CVD risk [6]. The gut produces TMA mostly from Firmicutes and Proteobacteria from choline and carnitine-rich foods. The production of TMA/TMAO has been linked to Deferribacteraceae, Anaeroplasmataceae, Prevotellaceae, and Enterobacteriaceae. Recent discoveries include eight TMA-forming bacteria from two phyla, Firmicutes and Proteobacteria [7]. *Clostridium asparagiforme* and *Escherichia fergusonii* produce TMA [8], which increases atherosclerosis (AS) risk [7]. Conversely, the intestines contain a wide range of probiotics. One probiotic that can prevent the growth of harmful bacteria in the intestines and safeguard the host’s health is *Bifidobacterium longum* [9].

Resveratrol (RSV) has been identified as a natural polyphenol stilbene in the human diet (e.g., in grapes, grape skins, red wine, apples, pistachios, plums, cranberries, cocoa, raspberries, strawberries, mulberries, peanuts, etc.) [10,11,12,13,14]. In addition, RSV served extensively in conventional medicine for a long time until 1997, first being reported to affect tumor initiation, advancement, and progression, followed by widespread reports of prophylactic and therapeutic potential due to its anti-oxidant, anti-aging, anti-inflammatory, anti-thrombotic, anti-obesity, anti-diabetic, anti-bacterial, anti-viral, anti-cancer, anti-atherosclerosis, cardioprotective, and immunomodulatory properties [13,15,16,17]. It is important to note that the RSV level found in red wines can vary significantly among different brands, and the quantity can range from 0.36 to over 2 mg/L, while it has been suggested to contribute to the French paradox [11,12,15,16,18,19]. Despite its trans-RSV-related compounds, 77–80% were absorbed from the gastrointestinal tract, and about 49–60% were eliminated via urine [20]. However, Tamargo et al. [21] reported a significant decrease in the bioaccessibility of cholesterol and bile salts during the digestion of lipids with wine using the simgi^®^ digestion system. Moreover, RSV has been shown to regulate gut microbial diversity; for example, it has contributed to the growth of metabolic trimethylamine (TMA)-producing *Bifidobacterium* and *Lactobacillus*, thereby reducing trimethylamine-*N*-oxide (TMAO) concentrations and alleviating TMAO-induced atherosclerosis [17,19,22,23,24]. However, in a typical case, 95–96% of TMA would be oxidized to TMAO, which is either transported to the tissues and accumulated or eliminated by the kidneys (excreted in urine) within 24 h or excreted in sweat, feces (4%), expired air (less than 1%), or other bodily secretions [25,26]. At the same time, non-hepatic tissues will absorb small amounts through FMO3 G472A polymorphism [26]. Notably, many studies have reported that broad-spectrum antibiotics nearly completely suppress intestinal microbes and the production of TMA and TMAO but that TMAO levels return to normal one month after discontinuing the antibiotics [25,27,28]. Expectedly, this recommendation for antibiotics in the regular population’s diet will not be recognized, while reducing the production of TMAO or inhibiting dietary sources of TMA presents an opportunity and challenge.

Glucosinolates in Brussels sprouts may prevent atherosclerosis [29]. Cashman et al. (1999) found that glucosinolate-rich Brussels sprouts inhibit human FMO3 [29]. Goitrin competes with FMO3’s active center to inhibit TMA oxidation to TMAO [30]. Iglesias-Carres et al. (2021) have suggested that phytochemicals with reduced TMAO bioactivity could act as prebiotics by modulating gut microbiota, directly inhibiting TMA lyase, or inhibiting hepatic FMO3 expression or activity [31]. While phytochemicals lower TMA and/or TMAO, they also have other cardioprotective effects. Thus, phytochemicals may protect the heart through multiple mechanisms, including TMAO reduction. This team previously published the synthesis of RSV and butyric acid, which resulted in a derivative, namely RBE, with similar bioactivity and better bioavailability [32]. Subsequently, an improved Steglich esterification reaction with N-ethyl-N′-(3-dimethyl aminopropyl) carbodiimide (EDC) and 4-dimethyl aminopyridine (DMAP) has been used with a 30% yield increase for RBE [33,34]. Regarding the currently known biological activities of REB, such as the H_2_O_2_ scavenging activity of REB, inhibition of fatty-acid-induced lipid accumulation in HepG2 cells [14] prevented liver damage caused by Bisphenol A (BPA) exposure during pregnancy, modulated of the gut microbiota on the gut–hepatic axis in offspring [35], inhibited BPA-induced obesity in female offspring rats [32], and prevented adenine-induced kidney damage and hypertension [36,37], while it inhibited lipid biosynthesis (it modulated adipogenic protein expression and increased the p-AMPK/AMPK ratio in 3T3-L1 adipocytes) [38].

How polyphenols like RSV and its derivatives can directly or indirectly lower TMA and TMAO levels in organisms is a major area of future research. The antioxidant, anti-obesity, and intestinal-bacteria-regulating properties of RSV and RSV derivatives akin to polyphenols, particularly pterostilbene, are of particular interest. Currently, there are some biological effects of RBE derivatives not appropriately understood. In addition, ED2 (di-butyric acid derivative) and ED4 (mono-butyric acid derivative) have been isolated from RBE with satisfactory bioactivities and a higher antioxidant capacity and potential compared to RBE [14]. RSV, as a natural polyphenol with prebiotic advantages, can alleviate TMAO-induced AS, affect the intestinal microbiota, and inhibit hepatic FMO3 expression or activity [31]. As stated above, this study hypothesized that RSV-derived products modified by butyric acid esterification may have the ability to improve regulation of the growth and decline of intestinal bacteria and the production of TMAO by liver cell metabolism. Therefore, this study aimed to identify RBE’s potential for biomedical applications by investigating its effects on proliferation within a co-culture of *C. asparagiforme* and *B. longum* and TMA content changes in the medium. In addition, this study also examined the benefits of RBE, ED2, and ED4 in HepG2 cell lines in terms of the potential for regulating TMA metabolism via the cells’ cellular model.

## 2. Results

### 2.1. Effects of the Mixed Medium on the Growth Curves of C. asparagiforme and B. longum

**Figure A1:**
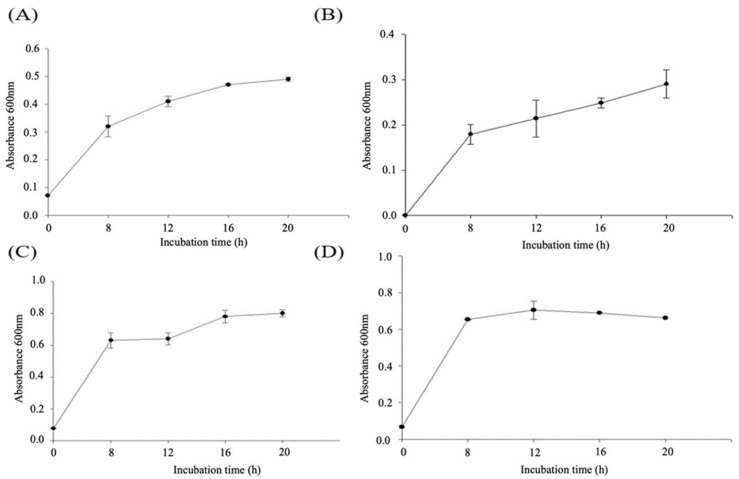
The growth curves for *C. asparagiforme* under (**A**) PY + X medium, (**B**) PY + X: MRS medium (1:1; *v*/*v*), (**C**) MRS medium, and (**D**) PY + X: MRS medium (1:1; *v*/*v*) medium against the influence of *B. longum*.

This study determined the effect of mixed medium (PY + X and MRS 1:1 ratio *v*/*v*) on the growth curves of *C. asparagiforme* and *B. longum*, respectively. The results showed that there were no significant differences between *C. asparagiforme* cultured in the recommended medium (PY + X) and mixed medium (Figure A1A,B); the growth curves for *B. longum* in the recommended medium (MRS) and mixed medium showed similar trends (Figure A1C,D). Therefore, according to this study’s results, the incubation time was set at 16 h because this was between the logarithmic and stabilization phases, which was advantageous for the subsequent trials.

### 2.2. Effects of Choline, RSV, and RBE Added to Medium against the Growth Curves of C. asparagiforme and B. longum

This study investigated the growth of *C. asparagiforme* (4% inoculum) incubated with different concentrations of choline (0, 0.1, 0.5, and 1%), REV (5, 50, and 100 µg/mL), and REB (5, 50, and 100 µg/mL), which revealed that all groups’ growths were influenced by 0.5 and 1.0% of choline (*p* < 0.05) (Table 1). However, this was considered to imply that choline inhibited *C. asparagiforme* growth; thus, the choline addition’s concentration was limited to 0.1% in subsequent trials. Moreover, the growth effects on *C. asparagiforme* in the RSV and RBE groups were negatively correlated with the concentrations of RSV and RBE (*p* < 0.05), particularly for RSV 100 µg/mL at 0.5–1.0% choline, RBE 50 µg/mL at 0.1–1.0% choline, and RBE 100 µg/mL (with or without choline) (*p* < 0.05). In the absence of choline, RSV of 5 or 100 µg/mL might promote *C. asparagiforme* growth. Although there was no significant difference to the other groups, there was an increase in the values. In contrast, choline, RSV, and RBE exhibited minor and partially statistically significant (*p* < 0.05) effects on *B. longum* growth (Table 1), while, overall, the differences in values were marginal. It is worth mentioning that RBE 5 µg/mL at 0.1–1.0% choline showed a tendency to promote the growth of *B. longum*, which was significantly different from all groups (*p* < 0.05).

**Figure 1:**
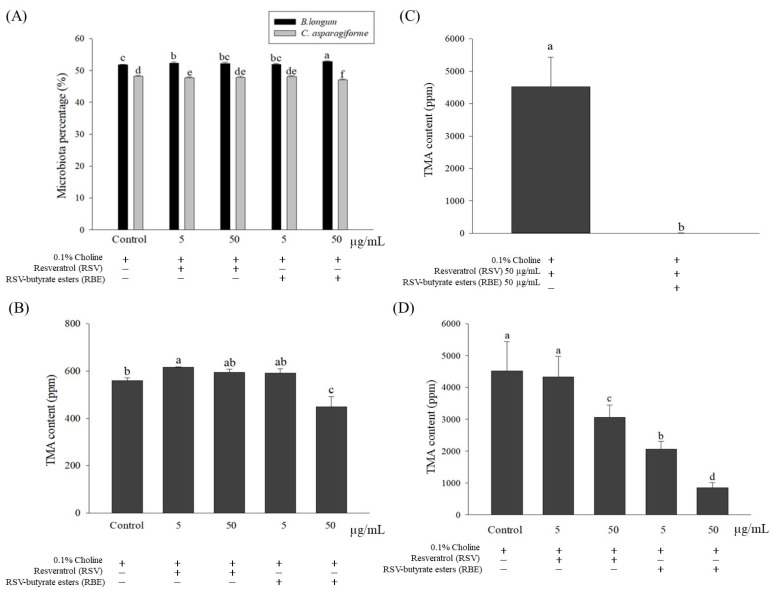
Effects of resveratrol (RSV) and RSV-butyrate esters (RBE) on bacterial cultures: (**A**) effects on the growth of *C. asparagiforme* and *B. longum* expressed as bacterial population percentage (%); (**B**) effects on the metabolic choline production of trimethylamine (TMA; ppm) by *C. asparagiforme*; (**C**) effects for a single culture of *C. asparagiforme* or co-culture of *C. asparagiforme* and *B. longum* of choline metabolized into TMA (ppm); and (**D**) effects of RSV and RBE treated with 50 μg/mL each on the metabolic choline yield of TMA in a single culture of *C. asparagiforme* and a co-culture of *C. asparagiforme* and *B. longum*. Different letters indicate that the statistically different from other (*p <* 0.05).

Subsequently, the effects of the above-optimized concentrations of choline (0.1%), RSV (5 µg/mL and 50 µg/mL), and REB (5 µg/mL and 50 µg/mL) on the growth of *C. asparagiforme* and *B. longum* (Figure 1A) were identified. This study revealed that both RSV and REB added slightly inhibited *C. asparagiforme* and promoted *B. longum* growth, which was significantly different (*p* < 0.05) compared to the control group.

### 2.3. Effect of Single Culture and Co-Culture on TMA Content Arising from Metabolized Choline

This study determined the effects of RSV and RBE on the TMA produced from metabolic choline by *C. asparagiforme.* It was shown that 50 µg/mL RBE in a 0.1% choline incubation environment could significantly reduce TMA content (Figure 1B), which was significantly different (*p* < 0.05) compared to all groups. Moreover, co-culture was conducted in this study, which revealed that TMA produced from metabolized choline by *C. asparagiforme* was also significantly inhibited (*p* < 0.05) in the case of probiotic (*B. longum*) presence (Figure 1C). Meanwhile, both results confirmed that *C. asparagiforme* metabolizes 0.1% choline and produces TMA, which was inhibited by 50 µg/mL RBE and *B. longum* (in vitro).

Subsequently, this study evaluated the variation in TMA content in the co-culture of both bacteria combined with the extra addition of RSV or RBE. This study showed that the extra RSV (50 µg/mL) and RBE (5 and 50 µg/mL) added under co-incubation were effective for the inhibition of TMA (*p* < 0.05) (Figure 1D), which was dose-dependent, and that RBE at 50 µg/mL performed satisfactorily (*p* < 0.05).

### 2.4. Evaluation of the Optimum Concentrations of Various Samples for the Effects of RBE and Its Ester Derivatives ED2 and ED4 on HepG2 Cell Lines’ Viability

**Figure 2:**
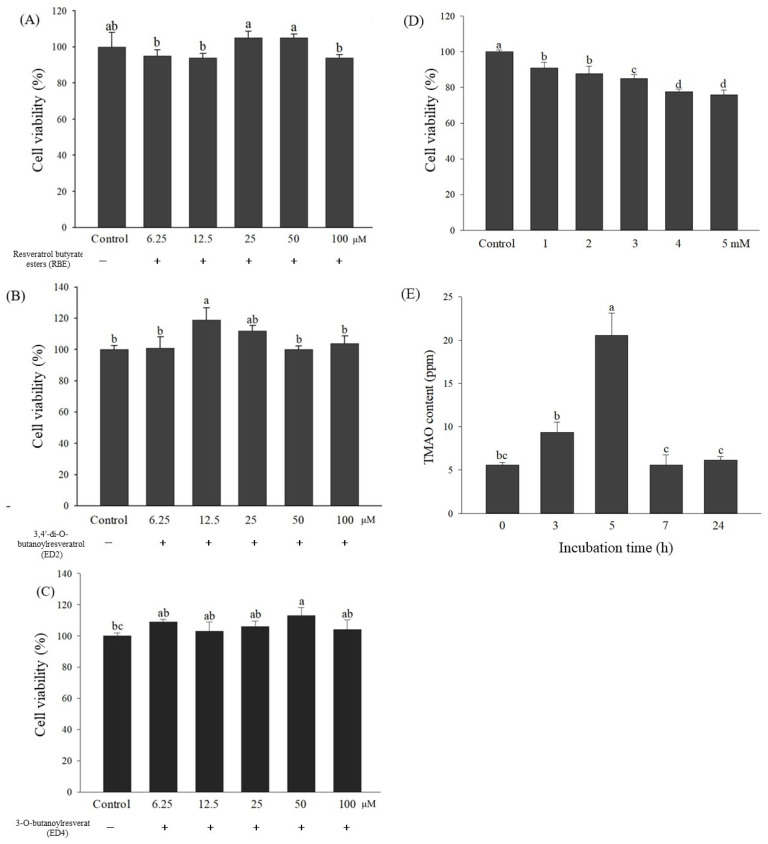
Effects of different concentrations of samples on HepG2 cell lines treated with (**A**) resveratrol butyrate esters (RBE); (**B**) 3,4′-di-O-butanoylresveratrol (ED2, di-butyric acid derivative); (**C**) 3-O-butanoylresveratrol (ED4); and (**D**) trimethylamine (TMA). (**E**) Effects of TMA treatment at different times on the amount of trimethylamine oxide (TMAO) produced by HepG2 cell lines. Different letters indicate that the statistically different from other (*p <* 0.05).

This study was conducted on the optimum RBE, ED2, and ED4 concentrations for HepG2 cell line cell viability. The results showed that treatment of HepG2 cell lines with the three samples at 6.25, 12.5, 25, and 50 µM for 24 h had no effect on cell viability compared to the control group (Figure 2A–C), despite the significant differences between the groups (*p* < 0.05). It is worth mentioning that RBE (25 and 50 µM), ED2 (12.5 and 25 µM), and ED4 (6.25 and 100 µM) exhibited proliferation of the HepG2 cell lines at several concentrations. However, considering the variable factor of concentration, the doses for the follow-up trials in this study were based on the maximum effective dose allowed for RBE, 50 µM. However, in terms of the different concentrations (1, 2, 3, 4, and 5 mM) in TMA treatment, the results showed that the viability of HepG2 cell lines in all TMA groups exhibited a decreasing trend, which showed a significant difference (*p* < 0.05) compared with the control group (Figure 2D). At the same time, they also implied the dose-dependence of TMA treatment on HepG2 cell line viabilities, among which 4 and 5 mM were decreased and reached below 80%. Therefore, 4 mM of TMA was applied in this study as an optimum concentration for subsequent trials. Subsequently, 4 mM TMA was used to investigate the effects of various treatment times on the TMAO contents produced by HepG2 cell lines metabolizing TMA. This study showed that TMAO content increased during 0–5 h of TMA treatment (*p* < 0.05) (Figure 2E), where 5 h treatment showed a nearly fourfold increase in TMAO compared to 0 h. However, a sharp decrease was exhibited at 7 h treatment, and the level remained stable until 24 h (*p* < 0.05).

The timing of mRNA expression is indeed closely related to the timing of protein translation and post-transcriptional modification. mRNA (messenger RNA) is a molecule that translates information in genes from DNA into protein. The following are some important processes related to the timing of mRNA expression: transcription, translation, and post-transcriptional modification. The timing of these processes is important because they influence the final function of the gene. In cells, these processes are often tightly regulated to ensure that the right proteins are synthesized at the right time and place.

**Figure 3:**
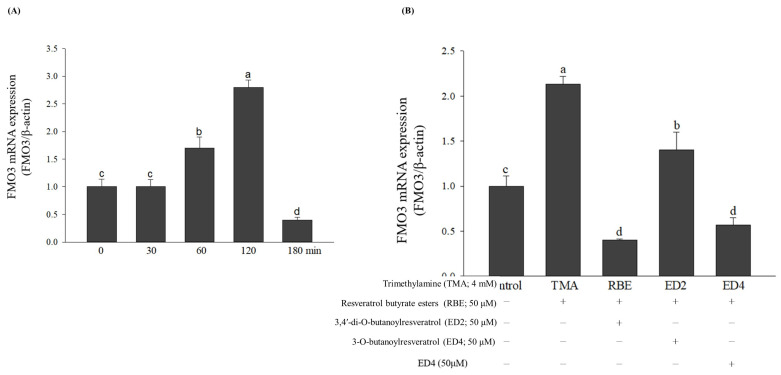
Effects of the different samples treated on flavin-containing monooxygenase-3 (FMO3) mRNA gene expression in the HepG2 cell line: (**A**) trimethylamine (TMA) treatment for 0–180 min; (**B**) resveratrol butyrate esters (RBE), 3,4′-di-O-butanoylresveratrol (ED2, di-butyric acid derivative), 3-O-butanoyl resveratrol (ED4), and TMA treatment for 180 min. Different letters indicate that the statistically different from other (*p <* 0.05).

In this study, it can be seen that FMO3 mRNA expression significantly decreased between 120 and 180 min of sampling (Figure 3A). At the same time, the sampling time for cell metabolism of TMA to TMAO peaked at 5 h and dropped significantly at 7 h (Figure 2E). The induction of FMO3 mRNA is affected by different stimuli and conditions, such as specific drugs, nutrients and environmental factors. The experimental data can obtain the optimal induction time for FMO3 mRNA, which means that appropriate gene expression can be well observed under specific conditions of drug treatment [39].

### 2.5. Effects of Various Treatments on FMO3 mRNA and Its Gene Expressions, Which Regulate TMA Metabolism in HepG2 Cell Lines

This study determined FMO3 mRNA expression to identify the FMO3 gene that regulates TMA metabolism in HepG2 cell lines. The results showed that the FMO3 mRNA gene expression showed an increasing trend as treatment time increased, which was significantly different for each group (*p* < 0.05) (Figure 3A), then decreased dramatically at 180 min (*p* < 0.05). In addition, this study used RBE, ED2, and ED4 cultured (180 min) with HepG2 cell lines. Rudraiah et al. (2014) reported that FMO3 expression and function protect the liver from acetaminophen (APAP)-induced toxicity. Although the mechanism of this protection remains to be elucidated, the work describes a novel protective function of this enzyme [40]. Rudraiah et al. (2014) is the first report describing a novel protective function for this drug-metabolizing enzyme [40]. FMO3 is one of several noteworthy genes differentially expressed in APAP self-protective mice. FMO3 is unique in that it is considered non-inducible [41], but in the self-protective group reported by O’Connor et al., FMO 3 mRNA expression increased 20-fold at 4 h and 7-fold at 24 h [42]. It is not clear what role FMO3 plays in APAP liver damage, but, from a biological point of view, the fact that it increases gene expression and shows metabolic enzyme activity makes it most likely that it plays a part in self-defense. In other words, the timing of induction of FMO3 mRNA depends on the specific context and study conditions. In this study, the peak induction of FMO3 in hepatocytes by TMA was at approximately 120 min. The results show that the RBE and ED4 groups can significantly inhibit FMO3 mRNA expression in HepG2 cell lines, whereas ED2 was third (*p* < 0.05) (Figure 3B).

## 3. Discussion

In its human circulation, the gut microbiota initially synthesizes TMA from dietary quaternary amines (e.g., choline, phosphatidylcholine, carnitine, betaine, and ergothioneine), followed by oxidation via flavin-containing monooxygenases (FAO) to form a small, odorless molecule called TMAO [25,43,44]. This study used the co-culture of *C. asparagiforme* (the strain would produce TMA under choline supplementation of the medium [7,45]) and *B. longum* to simplify the simulation of intestinal conditions, revealing that RSV and RBE at 5–50 µg/mL could inhibit *C. asparagiforme* growth. Therefore, it is hypothesized that the catabolic choline production of TMA would be effectively interrupted in this case, since *C. asparagiforme* growth was influenced. Despite Romano et al. [7], dietary choline in gnotobiotic mouse models appeared to provide no advantage for the growth of these choline-degrading TMA-producing strains (*C. hathewayi*, *P. rettgeri*, and *C. asparagiforme*).

Moreover, FMO3 represents the latest step in host-mediated TMAO production by the microbiome after it has metabolized the dietary nutrients; at the same time, the FMO enzyme family has been reported to be highly expressed in the liver [44]. Bennett et al. [46] reported that FMO3 mRNA levels were expressed more in the liver of female rats than in males. In humans, there were significant differences between males and females for FMO1 and FMO3, where FMO3 contributed over 90% of the total hepatic activity involved in TMA oxidation to TMAO. Moreover, Chen et al. [47] reported eight-fold increased plasma TMAO levels in C57BL/6J mice under a lithogenic diet (containing 1.25% cholesterol and 0.5% cholic acid), which was attributed to significantly increased expression of hepatic FMO3 mRNA inducing the catalysis of gut-derived TMA to circulating TMAO. In contrast, the liver also showed considerably increased TMAO levels. Trimethylamine-n-oxide, a metabolite associated with atherosclerosis, exhibits complex genetic and dietary regulation [17]. It is reported that dietary RSV supplementation facilitated the metabolism of TMA in the livers of C57BL/6J mice fed choline (400 mg/kg body weight) for 4 h while decreasing their TMAO level and reshaping the mice’s gut microbiota over a month-long feeding period. Similar results were found in another animal study via dietary methionine restriction, namely via the gut-microbiota-driven TMA/FMO3/TMAO pathway [48,49]. It is worth mentioning that the addition of RBE, ED2, and ED4 for the TMA-containing culture of HepG2 cell lines in this study also confirmed the downregulation of FMO3 gene expression by RBE and ED4. The development of cardiometabolic diseases is correlated with the production of TMAO through the gut microbial metabolism of TMA, which has also been implicated in cardiometabolic diseases concerning vascular inflammation (MAPK and NF-κB signaling pathways), oxidative stress, relaxation of endothelium-derived hyperpolarization, impairment of prostaglandin I_2_ secretion, and other triggered effects [50]. Unfortunately, Griffin et al. [51] reported that a six-month Mediterranean-style dietary intervention was insufficient to reduce TMAO levels in a healthy population, and the authors recommended that interrupting the dietary conversion of TMA to TMAO should be considered. However, some scholars have proposed that species differences affecting gut microbiota composition may explain the findings [52]. Moreover, the short-term interventions presented in animal models are insufficient to induce remodeling of the gut microbiota formed by years of habituation; instead, dysbiosis occurs and increases TMAO levels [52,53]. Interestingly, probiotic/prebiotic applications performed in human clinical and animal models effectively enhanced the abundance of probiotics in the intestines and significantly reduced TMA and TMAO metabolism, accompanied by other benefits such as decreased body weight, lipids, and cholesterol marker levels [52,54,55]. Probiotic supplementation alone completes the regulation of the gut microbiota, with improved lipid profiles and reduced inflammation [56,57,58,59].

However, considering the above considerations and improvements, this study showed that RBE, ED4, and a co-culture of *C. asparagiforme* and *B. longum* were influential in reducing choline metabolism to TMA in vitro. Despite the in vitro limitations of cross-species trials where anaerobic bacteria cannot be cultured with cell lines, the combination of the two approaches might help interrupt the conversion of TMA to TMAO. Thus, animal models and human clinics must provide more validation of the combination of REB, ED4, and probiotics against dietary-sourced TMA, including interruption and modulation mechanisms (such as the inhibition of FMO3 and TMAO by insulin [60]).

## 4. Materials and Methods

### 4.1. Materials

Trans-RSV, dimethyl sulfoxide (DMSO), TMA, chloroform, 2-mercaptoethanol, and (3-(4,5-dimethylthiazol-2-yl)-2,5-diphenyltetrazolium bromide (MTT) were purchased directly from Sigma-Aldrich^®^ (Merck KGaA, Darmstadt, Germany). Penicillin–streptomycin and 0.5% trypsin-EDTA were purchased from Gibco (Thermo Fisher Scientific Inc., Waltham, MA, USA). Human liver cancer cell lines (RM60025), *C. asparagiforme* (BCRC 80850), and *B. longum* subsp. *longum* (BCRC 14664) were purchased from the Bioresource Collection and Research Center (Hsinchu, Taiwan). ED2 and ED4 were the primary ester derivatives of RBE, and three samples were obtained by this team from the isolation of a previous study [14].

### 4.2. Activation and Cultivation of Bacteria

*C. asparagiforme* was cultured in PY + X medium modified from Mohan et al. [61], with 4% of the bacterial quantity in 10 mL PY + X medium, incubated anaerobically at 37 °C for 24 h for primary activation. The above operations were repeated for secondary activation. Each generation of cultivation was performed in the same way. In addition, *B. longum* subsp. *longum* was cultured in MRS medium modified from Yasmin et al. [62], inoculated with 1% bacteria into 10 mL medium, and then treated as described above. All incubations were carried out in anoxic environments using methods borrowed from Hungate and Bryant [63,64], using a gas phase of 80:20 by volume of N_2_ and CO_2_ for cultivation.

### 4.3. Choline, RSV, and RBE Addition and Bacterial Culture

After secondary activation, 400 μL of *C. asparagiforme* was incubated into 10 mL quantities of PY + X medium containing 0%, 0.1%, 0.5%, and 1% choline and then cultured in an anaerobic incubator at 37 °C for 24 h. Finally, 200 μL of the bacterial broth was transferred to a 96-well plate, and an ELISA reader (Bio Tek Epoch2, Agilent Technologies, Inc., Santa Clara, CA, USA) measured the absorbance at 600 nm. Again, 400 μL of bacteria broth was cultured into 10 mL quantities of PY + X medium containing 0 ppm, 5 ppm, 50 ppm, and 100 ppm of RSV and RBE, followed by the above steps. RSV and RBE were dissolved in DMSO, respectively, as 0.1% (*v*/*v*) of the total medium.

*B. longum* (20 μL) was cultured into 2 mL of MRS medium with choline (0%, 0.1%, 0.5%, and 1%), RSV (0 ppm, 5 ppm, 50 ppm, and 100 ppm), and RBE (0 ppm, 5 ppm, 50 ppm, and 100 ppm), respectively, which were anaerobically cultivated for 16 h at 37 °C. Subsequently, the determination was performed according to the above operations.

For co-culture, 20 μL of each strain was inoculated into 2 mL of PY + X: MRS (1:1; *v*/*v*) medium with 0.1% choline and then anaerobically cultured at 37 °C for 16 h, followed by measurement as described above.

### 4.4. Determination of Trimethylamine (TMA) and Trimethylamine-N-Oxide (TMAO) Contents in Bacterial Co-Cultured Medium

TMA and TMAO contents were determined as described by Hsu et al. [65] and Romano et al. [7], with some modifications. The above co-cultured medium was collected and centrifuged for 5 min (12,000× *g*, 4 °C), and then the supernatant was collected before addition of the internal standard (diethylamine). Next, the determination was performed using a Triple Quadrupole mass spectrometer (Agilent 6410, Agilent Technologies, Inc.) equipped with an electrospray ion source. In addition, the multiple reaction monitoring mode was set up (*m*/*z* of 60.1 → 44.1 and 76.1 → 58.1, respectively) to detect the contents of TMA and TMAO as ppm in the medium. Then, chromatographic separation was performed using an Agilent Technologies 1200 HPLC system with the following conditions: the separation was performed on a SeQuant ZIC-HILIC column (150 × 2.1 mm, 5 µm, Merck KGaA) and the protection column was an Ascentis (2 cm × 4 mm, 5 µm; Merck KGaA). Mobile phase A was methanol and ammonium formate (15 mmol/L), while mobile phase B was acetonitrile with a ratio of 20:80 at a flow rate of 0.3–1 mL/min.

### 4.5. HepG2 Cell Line Culture and Its Viability Assay

HepG2 cell lines were cultured in Dulbecco’s modified Eagle’s medium (DMEM; Merck KGaA) with 10% fetal bovine serum (FBS) (Cytiva, Marlborough, MA, USA) and cultured at 37 °C with 5% CO_2_ as described by [14]. In addition, cell viability was determined by MTT as described in Huang et al. [66] and Shih et al. [14] with minor modifications. HepG2 cell lines at 5 × 10^3^ cells/mL were inoculated into a 96-well plate; after 24 h of attachment, the cells were treated with media containing different concentrations (6.25, 12.5, 25, 50, and 100 µM) of RBE, ED2, ED4, and TMA (1, 2, 3, 4, and 5 mM) for 24 h. Then, to each well was added 20 µL of phosphate-buffered saline (PBS) containing 5 mg/mL MTT, and incubation was continued for 4 h. Next, 150 µL of DMSO was added after removing the medium to dissolve MTT–formazan crystallites, followed by absorbance measurement at 570 nm with an ELISA reader, while the following formula calculated the cell viability:

(1) $$Cell\;viability\left(\%\right)=\frac{Absorbance\;570\;nm\;of\;Sample}{Absorbance\;570\;nm\;of\;Control}\times 100$$

### 4.6. Determination of TMAO Content in Cell Line Media

This study was performed using the Human TMAO ELISA Kit (MBS7269386, MyBioSource, Inc., San Diego, CA, USA) and measured following the standard operating procedures provided by the manufacturer. The reaction was performed in 100 μL of medium or standard in a 96-well plate, followed by adding 50 μL of reactive enzyme and protecting the response of light for 1 h. Next, the solution in each well was removed and washed 5 times with wash solution. Then, 50 μL of substrates A and B were added, respectively, followed by a reaction at 37 °C for 20 min. Finally, 50 μL of stop solution was added, and, immediately, the absorbance was measured at 450 nm with an ELISA reader. The TMAO content in the cell culture medium was calculated by interpolation from the standard curve of the traditional preparation.

### 4.7. Expression Analysis of Relevant Metabolic Genes in Cell Lines

#### 4.7.1. Determination of Total RNA Content

This study used a total RNA isolation reagent kit (Cat: NA003-0100, GenDireX, Inc., Hsinchu, Taiwan) for isolation and followed the operational procedures provided by the maker. The culture plate was put in an iced bath, then the medium was removed and washed 3 times with 1× PBS. Next, 500 μL of GR Buffer 1 and 8 μL of β-hydroxy ethanethiol were added, and the reaction was performed in a dry bath heater at 60 °C for 10 min. Then, 58 μL of GR Buffer 2 and 500 μL of chloroform were added, shaken vigorously, and centrifuged for 10 min (4 °C, 14,000× *g*). The supernatant was transferred to a new microcentrifuge tube, mixed with equal isopropanol, and reacted for 10 min in an ice bath. Next, the material was centrifuged again for 15 min (4 °C, 14,000× *g*), the supernatant was removed, and 1 mL of 70% ethanol was added to wash the residue, followed by centrifuging for another 5 min (4 °C, 14,000× *g*). Subsequently, the supernatant was removed, and 100 μL of RNase-free H_2_O was added to dissolve the residues, which were reacted in a dry bath heater at 60 °C for 10 min, quantified, and stored at −80 °C. It is important to note that all of the equipment mentioned above was sterilized (121 °C, 20 min) to remove the RNase remaining on the articles. Total RNA was quantified with a spectrophotometer by determining the absorbances at 260 nm and 280 nm, respectively, calculating the ratio (ABS_260nm_/ABS_280nm_) as the concentration of the sample and the quality of the RNA, and confirming that the ratio was between 1.9–2.1 before performing the next step.

#### 4.7.2. Total RNA Reverse Complement to DNA

This study was performed using a GScript first-strand synthesis kit (Cat: MB305-0050, GenDireX, Inc.) following the protocol provided by the manufacturer. Briefly, for the first cDNA synthesis, 1 μL of 50 μM oligo (dT)_20_ and 10 mM dNTPmix and then sterile water was added to reach a total volume of 13 μL in a microcentrifuge tube. The tube was then placed in a dry bath heater at 65 °C for 5 min followed by oscillation and placed in an ice bath for 5 min. Then, 4 μL of 5 × 1st strand buffer, 1 μL of 0.1 M DTT, 0.25 μL of RiboINTM RNase inhibitor, and 1 μL of GScript RTase (200 U/μL) were added sequentially, followed by quantification to 20 μL of the total volume with sterile water, mixing uniformly. Next, the sample was heated in a dry bath heater at 50 °C for 1 h, switching to 70 °C for 15 min to obtain the first cDNA solution, which was kept at −20 °C until used.

#### 4.7.3. Real-Time Polymerase Chain Reaction (RT-PCR)

For quantitative analysis, 5 μL of 2 × SYBR fast master mix (Q411-02, Topgen Biopharm Co., Shanghai, China), 0.5 μL of 20 × Target primer set (600119, Topgen Biopharm Co.), 2.5 μL of distilled deionized H_2_O, and 2 μL of cDNA (10 ng) were sequentially added, for a total volume of 10 μL. Next, the reaction was performed using an RT-PCR instrument (LightCycler^®^96 System, Roche Diagnostics, Rotkreuz, Switzerland), and detailed setup conditions are shown in Table A1. In addition, the control group in this study was human actin (β-actin), and the gene-specific primers used are shown in Table A2.

### 4.8. Statistical Analysis

All trials in this study were performed at least three times, with three samples analyzed per trial, and all the values are expressed as mean ± standard deviation (SD). All data were statistically analyzed with IBM SPSS software (version 22, Armonk, NY, USA). The differences between groups were analyzed using one-way variance (ANOVA), while Duncan’s *t*-test and the *t*-test were used to analyze the differences between all groups. The differences were considered significant at *p* < 0.05.

## 5. Conclusions

This study used a co-culture model to demonstrate how RSV and its derivatives of butyric acid can influence *B. longum* and impede *C. asparagiforme* development. This results in a decrease in metabolic choline, lowering TMA production. Moreover, this study augmented the inhibition of TMA by extra supplementation with RSV (50 µg/mL) and RBE (5 and 50 µg/mL), which was best performed with RBE supplemented at 50 µg/mL. It is also worth mentioning that both RBE and ED4 decreased FMO3 gene expression in cellular patterns significantly in the induction of TMA, which was hypothesized to have the potential to interrupt the TMO/FMO3/TMAO metabolic pathway and decrease TMAO. Hence, conducting a meticulous investigation through animal or clinical trials is imperative to assess the combined utilization of both techniques. All of the information provided so far in this study will be helpful for researchers in related fields applying theoretical guidance in practice to understand the complex interactions of intestinal flora with metabolic capacity, especially the pathways of the dietary sources of TMO metabolism to TMAO, with a positive assessment that will contribute to dietary and health promotion while creating opportunities in the future food supplement market.

## Figures and Tables

**Table 1:** Effects of *C. asparagiforme* and *B. longum* on the growth of different concentrations of resveratrol or resveratrol butyrate corresponding to different concentrations of choline.

Bacterial Strain	Choline (%)	Control	Resveratrol (RSV; µg/mL)			RSV-Butyrate Esters (RBE; µg/mL)		
			5	50	100	5	50	100
*C. asparagiforme*	0	0.30 ± 0.03 ^Ba^	0.32 ± 0.02 ^Ba^	0.27 ± 0.06 ^Bb^	0.32 ± 0.01 ^Ba^	0.28 ± 0.01 ^Cb^	0.29 ± 0.01 ^Bb^	0.07 ± 0.01 ^Cc^
	0.1	0.28 ± 0.00 ^Ba^	0.25 ± 0.01 ^Ba^	0.24 ± 0.00 ^Bb^	0.21 ± 0.02 ^Cc^	0.23 ± 0.01 ^Cc^	0.11 ± 0.00 ^Cd^	0.07 ± 0.00 ^Cd^
	0.5	0.10 ± 0.00 ^Ca^	0.09 ± 0.00 ^Ca^	0.09 ± 0.00 ^Ca^	0.11 ± 0.00 ^Da^	0.08 ± 0.00 ^Da^	0.07 ± 0.00 ^Ca^	0.07 ± 0.00 ^Ca^
	1	0.08 ± 0.00 ^Ca^	0.08 ± 0.00 ^Ca^	0.09 ± 0.01 ^Ca^	0.10 ± 0.00 ^Da^	0.08 ± 0.00 ^Da^	0.07 ± 0.00 ^Ca^	0.06 ± 0.00 ^Ca^
*B. longum*	0	0.57 ± 0.01 ^Ab^	0.55 ± 0.00 ^Ab^	0.53 ± 0.03 ^Ab^	0.46 ± 0.02 ^Ac^	0.64 ± 0.05 ^Aa^	0.56 ± 0.02 ^Ab^	0.52 ± 0.02 ^Ab^
	0.1	0.55 ± 0.01 ^Aa^	0.57 ± 0.00 ^Aa^	0.48 ± 0.01 ^Ac^	0.49 ± 0.03 ^Ac^	0.57 ± 0.02 ^Ba^	0.53 ± 0.01 ^Ab^	0.56 ± 0.03 ^Aa^
	0.5	0.55 ± 0.01 ^Aa^	0.54 ± 0.01 ^Aa^	0.50 ± 0.02 ^Ab^	0.45 ± 0.02 ^Ac^	0.55 ± 0.02 ^Ba^	0.51 ± 0.01 ^Ab^	0.51 ± 0.01 ^Ab^
	1	0.51 ± 0.01 ^Aa^	0.53 ± 0.01 ^Aa^	0.49 ± 0.03 ^Aab^	0.43 ± 0.03 ^Ac^	0.54 ± 0.03 ^Ba^	0.52 ± 0.01 ^Aa^	0.47 ± 0.02 ^Bb^

Data are presented as mean ± SD, *n* = 3. Different uppercase letters in the same column represent significant differences (*p* < 0.05). Different lowercase letters in the same row represent significant differences (*p* < 0.05).

## Data Availability

Data are contained within the article.

## Appendix A

**Table A1:** Conditions of real-time PCR analysis.

Program	Temperature (°C)	Time (s)	Cycle
1	95	60	1
	95	15	
2	49	60	40
	95	10	
3	65	60	1
	97	1	

**Table A2:** Specific primers in real-time PCR analysis in this study. F: forward primer. R: reverse primer.

Gene	Primer Sequence (5′-3′)	Reference (NCBI GenBank)
β-actin	F-CATCCGCAAAGACCTGTACG R-CCTGCTTGCTGATCCACATC	KR_710455.1
FMO3	F-CTCCCAGCAAGCATTCTGTG R-CATCAAGGAAGGGGTAGGCA	KR_712216.1
